# Good on the Belly-rippers

**Published:** 1891-08

**Authors:** 


GOOD ON THE BELLY-RIPPERS.—One of the country practitioners from one of the upper counties in this State was recently on a visit to New York, and among the other wonders of Gotham took in the Polyclinic. It was one of Wylie's field days, who, at the conclusion of a brilliant clinic, asked Dr. F. "what he thought of medical matters in the metropolis." Dr. F. replied: "Well, I would rather be a moonshiner down in Tennessee than a uterus up here in the hands of you New York doctors." —*St. Louis Medical and Surgical Journal.*

